# Toxicity of Flonicamid to *Diaphorina citri* (Hemiptera: Liviidae) and Its Identification and Expression of Kir Channel Genes

**DOI:** 10.3390/insects15110900

**Published:** 2024-11-18

**Authors:** Jiangyue Zhu, Xinjing Wang, Yunfei Mo, Beibei Wu, Tuyong Yi, Zhongxia Yang

**Affiliations:** Hunan Provincial Key Laboratory for Biology and Control of Plant Diseases and Insect Pests, College of Plant Protection, Hunan Agricultural University, Changsha 410128, China; zhujiangyue0612@163.com (J.Z.); wxj000501@163.com (X.W.); moyf0531@163.com (Y.M.); wubeibei095@163.com (B.W.)

**Keywords:** flonicamid, *Diaphorina citri*, transcriptome sequencing, inward-rectifying potassium channel, RT-qPCR

## Abstract

Flonicamid is a selective insecticide effective against piercing–sucking insects. Although its molecular target has been identified in other species, the specific effects and detailed mechanism of action in *Diaphorina citri* Kuwayama remain poorly understood. In this study, we determined that the LC_50_ of flonicamid for *D. citri* adults was 16.6 mg AI L^−1^ after 4 days of exposure. To explore the relevant mechanisms, the treatments with acetone and with 20 mg AI L^−1^ flonicamid for 96 h were collected as samples for RNA-Seq. The analysis of the transcriptomes revealed 345 differentially expressed genes (DEGs) in *D. citri* adults subjected to different treatments. Among these DEGs, we focused on the inward-rectifying potassium (Kir) channel genes, which have been extensively studied as potential targets of flonicamid. Three Kir subunit genes (*Dckir1*, *Dckir2*, *Dckir3*) in *D. citri* were successfully cloned and identified. Furthermore, the expression profiles of these *DcKirs* were investigated using RT-qPCR and showed that their expression significantly increased after *D. citri* eclosion to adulthood, particularly for *DcKir3*. The *DcKirs* were predominantly expressed in gut tissues, with *DcKir1* and *DcKir2* exhibiting high expression levels in the hindgut and midgut, respectively, while *DcKir3* showing high expression in the midgut and Malpighian tubules. This study provides insights into the potential roles of Kir subunits in *D. citri* and enhances our understanding of the physiological effects of flonicamid in this pest.

## Simple Summary

The Asian citrus psyllid, *Diaphorina citri*, the main insect vector for the spread of citrus Huanglongbing, has developed high levels of resistance to many commonly used insecticides. Flonicamid is a selective insecticide used for controlling piercing–sucking insects, but its toxicity and molecular target in *D. citri* are not well understood. This study found that flonicamid can be used as a new insecticide to control *D*. *citri*, likely by targeting inward-rectifying potassium channels (Kir) genes in the insect. We identified and cloned three Kir genes in *D. citri* and analyzed their expression in different tissues and life stages. These results provide a basis for further research on the insecticidal mechanism of flonicamid and contribute to improved methods to manage citrus pests.

## 1. Introduction

Flonicamid, a novel pyridine carboxamide insecticide developed by Ishihara Sangyo Kaisha, Ltd., in Japan, was first introduced to the U.S. market in 2003 [1]. It is a highly selective insecticide designed to target pests with piercing–sucking mouthparts, such as aphids, leafhoppers, whiteflies, and ticks [2,3]. Notably, flonicamid is non-toxic to beneficial insects, such as bees [4], parasitic wasps [5], ladybugs [6,7], and hunting bugs [1,8].

Research has demonstrated that flonicamid can effectively inhibit feeding in peach aphids by suppressing their sucking signals and salivation, ultimately leading to starvation and death of the pest [9]. The researchers investigated the insecticidal activity of flonicamid against *Chilo suppressalis* and found that the guts of the larvae shrunk after treatment with flonicamid, indicating that flonicamid inhibited the feeding of *C. suppressalis* [10]. Notably, flonicamid is not cross-resistant to any conventional insecticides currently in use, indicating a difference in its target of action [11]. Therefore, the target of flonicamid has become a popular research topic.

In a study of the rice pest, *Nilaparvata lugens*, entomologists found that flonicamid inhibited the inward rectifier potassium 1 (Kir1) channels at nanomolar concentrations, demonstrating that the Kir1 channel is the target of flonicamid [11]. However, subsequent research has offered a different perspective. A recent study identified nicotinamidase (Naam) as the actual molecular target of flonicamid, challenging the earlier hypothesis involving inward rectifier potassium (Kir) channels [12]. Nevertheless, these findings do not completely rule out the potential influence of flonicamid on Kir channels, particularly in different insect species and under varying physiological conditions.

Kir channels are a type of potassium channel that belongs to a family of channels that includes, Ca^2+^-activated (K_Ca_) potassium channels, voltage-gated (K_V_) potassium channels, two-pore structural domain (K2P) potassium channels, and cyclic nucleotide-gated (K_CNG_) potassium channels [13,14]. Insects are composed primarily of three classes of Kir (Kir1, Kir2, and Kir3), but recent studies have revealed the existence of a *Kir2* subtype in lepidopteran insects, initially proposed as *Kir4* [14,15]. Kir is distributed in almost all animal cells, but *Kir1* is highly expressed in the salivary glands and fat bodies of *N. lugens*, whereas *NlKir2* is highly expressed in the midgut, hindgut, Malpighian tubules, and salivary glands. Finally, *NlKir3* is mainly concentrated in the Malpighian tubules, followed by salivary glands, midgut, and hindgut [11]. Recently, researchers have demonstrated a strong interest in the role of Kir in physiological effects, toxicology, and pharmacology, but there has been less research on Kir in insects, so further work is needed to determine its expression pattern in this group.

The Asian citrus psyllid, *Diaphorina citri* Kuwayama (Hemiptera: Liviidae), the main insect vector for the spread of citrus Huanglongbing, has developed high levels of resistance to many commonly used insecticides. This resistance poses a significant challenge to controlling the spread of the disease [16,17,18]. Flonicamid is a highly effective selective insecticide that has been studied on *Amrasca biguttula* [19], *Nilaparvata lugens* [11], *Aphis glycines* [9,20], and other piercing–sucking mouthparts pests [1,5], but its efficacy on *D. citri* has not been reported. In this article, the toxicity of flonicamid to *D. citri* was investigated, and the transcriptome was comparatively analyzed after flonicamid treatment. Additionally, we identified the *DcKirs* and explored their specific expression pattern. The results establish a foundation for understanding the effects of flonicamid on piercing–sucking mouthpart pests and provide valuable information for further research on the Kir channel in Hemiptera.

## 2. Materials and Methods

### 2.1. Insects and Tissue Isolation

Adult *D. citri* populations were collected in 2022 from a wild citrus grove located in Chenzhou City, Hunan Province, China. They were reared in insect rearing cages (25 × 25 × 50 cm) on uninfected *Murraya exotica* L. at 25 ± 1 °C, 70% ± 5% relative humidity and a 16 h: 8 h dark: light cycle.

Tissue samples of *D. citri* at different developmental stages were collected using the following method: the first instar sample contained 100 nymphs, the second instar sample contained 50 nymphs, the third instar sample contained 40 nymphs, the fourth instar sample contained 20 nymphs, and the fifth instar sample contained 10 nymphs. In addition, each of the 1-day-old female or male adult samples contained 10 individuals, and every egg sample consisted of around 200 eggs. The 1-day-old *D. citri* adults (female:male = 1:1) were dissected, and their wings, legs, salivary glands, Malpighian tubules, midgut, hindgut, testes of male adults, and ovaries of female adults were extracted. The sample consisted of tissues from 100 individuals in each case.

### 2.2. Insecticide Exposure

The bioassay used *D. citri* adults in similar growth states as test insects. The insecticide flonicamid (97% purity) was provided by Shenzhen Noposion Agrochemicals Co., Ltd. (Shenzhen, China) and dissolved in acetone containing 0.1% of Tween 20 to form a stock solution. The stock solution was then diluted with 0.1% of Tween 20 to each gradient and the flonicamid was diluted to 40, 20, 10, 5, and 2.5 mg AI L^−1^. The control group was treated with acetone containing 0.1% of Tween 20.

The glass bottle walls were exposed to different concentrations of flonicamid. Young citrus leaves were dipped in the agent and the citrus petioles were moisturized with a moist cotton ball wrapped in tinfoil. Ten *D. citri* adults were placed in each bottle (with air holes) containing citrus leaves, with four replicates for each treatment. The number of deaths was recorded at 24, 48, 72, and 96 h after placing the adults into the bottles. Adults were considered dead if they did not show any sign of movement when they were touched with a small brush. Additionally, the guts of *D. citri* adults treated with flonicamid were dissected for microscopic observation.

### 2.3. RNA Isolation and Transcriptome Sequencing

The surviving *D. citri* adults treated with 20 mg AI L^−1^ of flonicamid were collected after 96 h and stored at −80 °C until assayed. Ten individuals of *D. citri* treated under the same conditions were considered one replicate, with acetone as the control, and three replicates performed for each treatment. RNA-seq analysis was conducted.

The Trizol reagent kit (Invitrogen, Carlsbad, CA, USA) and the Agilent 2100 Bioanalyzer (Agilent Technologies, Palo Alto, CA, USA) were used for the total RNA extraction and quality assessment. Additionally, RNase-free agarose gel electrophoresis was performed to check its quality. Following total RNA extraction, eukaryotic mRNA was purified using Oligo (dT) beads. Subsequently, the purified mRNA was broken into shorter fragments using a fragmentation buffer. The NEBNext Ultra RNA Library Prep Kit for Illumina (NEB #7530, New England Biolabs, Ipswich, MA, USA) was used to reversely transcribe the fragmented mRNA into cDNA. The double-stranded cDNA fragments were purified and prepared for sequencing by undergoing end repair, the addition of an A base, and ligation with Illumina sequencing adapters. To purify the ligation reaction, AMPure XP Beads (1.0X) were employed. Afterwards, the Ligated fragments were analyzed for size by using agarose gel electrophoresis and amplified using the polymerase chain reaction (PCR). The generated cDNA library was subjected to sequencing using Illumina Novaseq6000 by Gene Denovo Biotechnology Co., Ltd. (Guangzhou, China).

### 2.4. De Novo Assembly and Annotation of Transcripts

The transcript data were assembled and annotated by filtering low-quality raw sequencing reads using the analysis platform of Omicshare (Gene Denovo Biotechnology Co., Ltd., Guangzhou, China). The reference genome was downloaded from the *D. citri* genome database (https://citrusgreening.org/ftp/user_requests/surya/acp/ (accessed on 3 March 2023)). HISAT2. 2.4 (https://anaconda.org/biobuilds/hisat2 (accessed on 3 March 2023)) was utilized to construct an index of the reference genome and align the paired-end clean reads with the reference genome. The reads per kilobase per million reads mapped (RPKM) method was used to identify differentially expressed genes (DEGs), with the screening criteria of |log2 fold change| ≥ 1 and FDR < 0.1. The gene annotation process involved utilizing a blasting technique on multiple databases, such as NCBI protein non-redundant (NR), Gene Ontology (GO), and Kyoto Encyclopedia of Genes and Genomes (KEGG).

### 2.5. Cloning of D. citri Kir Genes

Total RNA was extracted from adults of *D. citri* using RNAiso Plus reagent (Takara Bio, Kusatsu, Shiga, Japan) according to the manufacturer’s instructions. The FastKing gDNA Dispelling RT SuperMix (TIANGEN, Beijing, China) was synthesized to generate cDNA templates from 1 μg total RNA. All products were stored at −20 °C until use. The reference genome was downloaded from the *D. citri* genome database (https://citrusgreening.org/ftp/user_requests/surya/acp/ (accessed on 29 March 2023)). Primers were specifically designed to amplify the complete coding sequence (CDS) of *Dckirs* based on the Kir sequences in *D. citri* genome database (Table S1). PCR was run using 2 × Phanta^®^ Max Master Mix (Dye Plus) (Vazyme, Nanjing, China), and the reaction was carried out as follows: 3 min at 95 °C; 35 cycles (15 s at 95 °C, 15 s at 55 °C, 90 s at 72 °C); and 5 min at 72 °C.

### 2.6. RT-qPCR Validation

The accuracy of RNA-Seq results was confirmed using RT-qPCR. cDNA template was synthesized with 1 μg total RNA and the HiScript II Q RT SuperMix for qPCR (+gDNA wiper) (Vazyme, Nanjing, China) according to the manufacturer’s instructions. The qRT-PCR analysis was conducted on a QuantStudio 1 Real-Time PCR System (Applied Biosystems, Thermo Fisher Scientific, Carlsbad, CA, USA) using ChamQ Universal SYBR qPCR Master Mix (Vazyme, Nanjing, China) at a 20 µL volume. qRT-PCR primers of candidate genes were designed using information from the National Center for Biotechnology Information profile server (http://www.ncbi.nlm.nih.gov/tools/primer-blast (accessed on 3 June 2023)) (Table S1). To determine the effectiveness of the qRT-PCR primers, we assessed their efficiency by creating a threefold dilution series of cDNA samples that corresponded to 1 µg of total RNA, and this was used to produce a standard curve (cDNA concentration vs. Ct). Each well plate was loaded with 1 µL cDNA. The qRT-PCR protocols were as follows: 95 °C for 30 s; 40 cycles of 95 °C for 10 s; and 60 °C for 15 s. To calculate the relative expression levels of each candidate gene, the arithmetic mean values of β-actin (GenBank: DQ675553) were used as the internal references [21]. Relative transcript levels of the target genes were calculated by the 2^−ΔΔCT^ method [22].

### 2.7. Statistical Analysis

GraphPad Prism 8.0.2 was used for graphing. The probit analysis of mortality data for each concentration was used to calculate the slopes, LC_50_ values, and 95% confidence limits for the toxicity of flonicamid against the *D. citri*, using SPSS 26.0 software (IBM, Chicago, IL, USA). The protein domains of Kir channels were predicted by SMART (https://smart.embl.de/ (accessed on 22 December 2023)). Multiple sequence alignment was carried out using Clustal W algorithm integrated in MEGA11. The phylogenetic trees were constructed with MEGA11 by using the neighbor-joining method. Bootstrap analysis was performed on 1000 replicate data sets to evaluate the significance of the nodes. The amino acid sequences of *DcKirs* were aligned by DNAMAN 6.0.3 (Lynnon Biosoft, San Ramon, CA, USA). All values are expressed as the mean ± SEM, and one-way ANOVA with LSD (Least Significant Difference) multiple comparison tests was used to assess the differences between the expression level of different stages and tissues of *DcKirs*, and statistical significance was established as *p* < 0.05. Differences in mRNA expression levels validated by RT-PCR after Flonicamid treatment were statistically analyzed using Student’s *t*-test.

## 3. Results

### 3.1. The Toxicity of Flonicamid to D. citri

**Figure 1:**
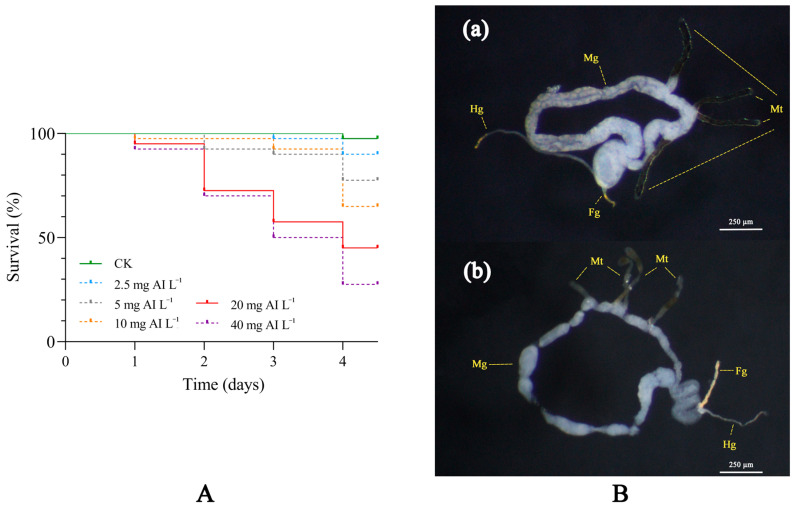
The toxicity of flonicamid on *D. citri* adults. (**A**) Survival curves of *D. citri* adults to different concentrations (2.5-40 mg AI L^−1^) of flonicamid for the first four days post treatment. (**B**) Changes in midgut and Malpighian tubules of *D. citri* adults after treatment with flonicamid. (**a**) The gut of *D. citri* adults treated with acetone. (**b**) The gut of *D. citri* adults treated with acetone and flonicamid (LC_30_). Tissue abbreviations: Malpighian tubules, Mt; foregut, Fg; midgut, Mg; and hindgut (Hg).

The toxicity of flonicamid to *D. citri* was determined using the residual film method after treatment with different concentrations (ranging from 2.5 mg AI L^−1^ to 40 mg AI L^−1^) for 4 days, and the mortality rate of the control group was lower than 3% (Figure 1A). The LC_50_ confidence intervals of *D. citri* treated with flonicamid for 4 days ranged from 12.5 to 23.9 mg AI L^−1^ (Table 1). These results suggest that flonicamid is effective against *D. citri* adults.

After 48 h of treatment with flonicamid, the gut of dying *D. citri* was observed under a microscope. Compared to the controls, the midguts of dying adults appeared more flaccid and shrunken, and the Malpighian tubules showed areas of uneven coloration. The plumpness of the gut was possibly related with the feeding status of the insects, which may suggest reduced feeding in adult *D. citri* (Figure 1B).

### 3.2. Illumina Sequencing, Read Assembly, and Annotation

We sequenced the transcriptome of *D. citri* adults treated with acetone or 20 mg AI L^−1^ flonicamid for 4 days. Each transcriptome produced more than 43 million raw reads, and the numbers of clean reads varied from 42,835,710 to 60,773,960 across the six transcriptomes. Both Q20 and Q30 showed a high-quality level, surpassing 97.5% and 93.3%, respectively (Table S2). Among clean reads, 84.2% to 85.7% of each transcriptome was successfully aligned to the genome of *D. citri*. Within these alignments, around 57.6% to 59.6% corresponded to exons, while 31.0% to 32.5% corresponded to introns (Table S3). Out of the combined sequences, a total of 21,378 unigenes were assembled, with 2329 of them being identified as novel genes.

### 3.3. Genes Affected After Flonicamid Exposure

**Figure 2:**
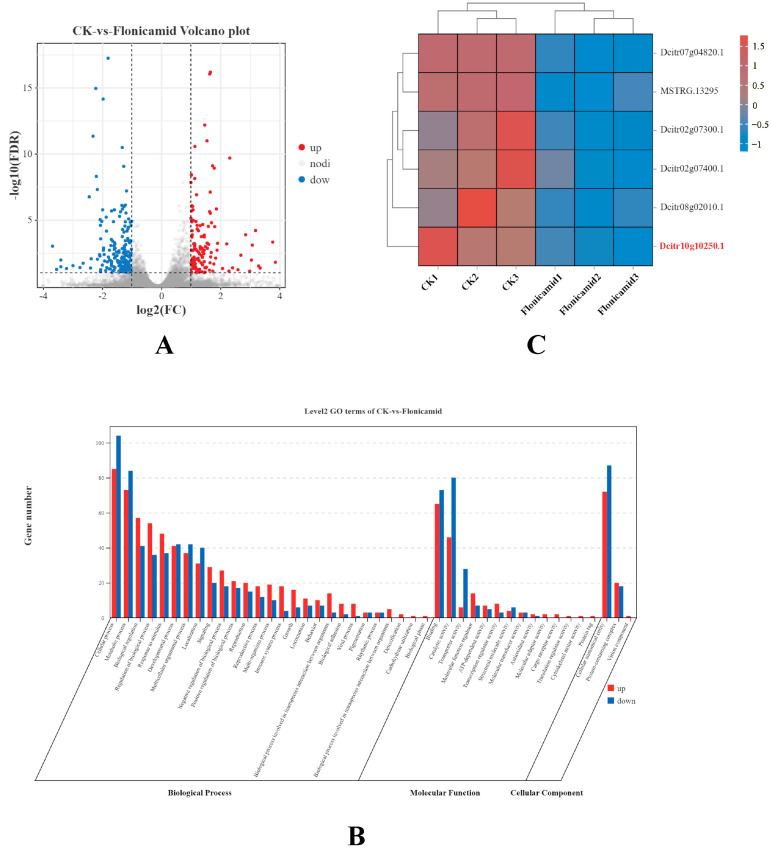
DEGs in *D. citri* after being treated with 20 mg AI L^−1^ flonicamid. (**A**) The volcano plots of DEGs identified from *D. citri* responsive to flonicamid treatments. Red spots: up-regulated DEGs; blue spots: down-regulated DEGs; gray spots: genes with no significant change in expression levels. (**B**) GO enrichment analysis of DEGs obtained from the comparison of flonicamid exposure and control group of *D. citri* adults. Red histograms: up-regulated DEGs; blue histograms: down-regulated DEGs. (**C**) The heat map indicates the DEGs related to ion channel activity after flonicamid exposure. A shift in color from red to blue indicates a change in the level of gene expression.

To identify the genes in *D. citri* adults that respond to flonicamid, the transcript levels of each gene were calculated using its FPKM value in different samples. A total of 345 genes were identified as DEGs between controls and adults treated with 20 mg AI L^−1^ flonicamid. Among them, 136 DEGs were up-regulated, while 209 DEGs were down-regulated (Figure 2A). The DEGs obtained from the comparison of Flonicamid exposure and control group were enriched in GO terms and classified by gene function: biological processes (cellular process and metabolic process); molecular functions (binding and catalytic activity); and cellular components (cellular anatomical entity and protein-containing complex) (Figure 2B).

Insect ion channels not only mediate information communication between cells, but also serve as targets for a variety of insecticides. In this study, we found that six genes involved in the activity of ion channels were affected by flonicamid. Surprisingly, all of the six DEGs were down-regulated, including genes sodium-independent sulfate anion transporter (Dcitr07g04820.1), voltage-dependent calcium channel subunit alpha-2/delta-4 isoform X2 (Dcitr02g07300.1), zinc finger protein 64-like (MSTRG.13295), open rectifier potassium channel protein 1 isoform X1 (Dcitr08g02010.1), inward rectifier potassium channel 2 (Dcitr10g10250.1), and voltage-dependent calcium channel subunit alpha-2/delta-4 isoform X2 (Dcitr02g07400.1) (Figure 2C).

### 3.4. Analysis of Genes Encoding in Inward Rectifier Potassium Channel

**Figure 3:**
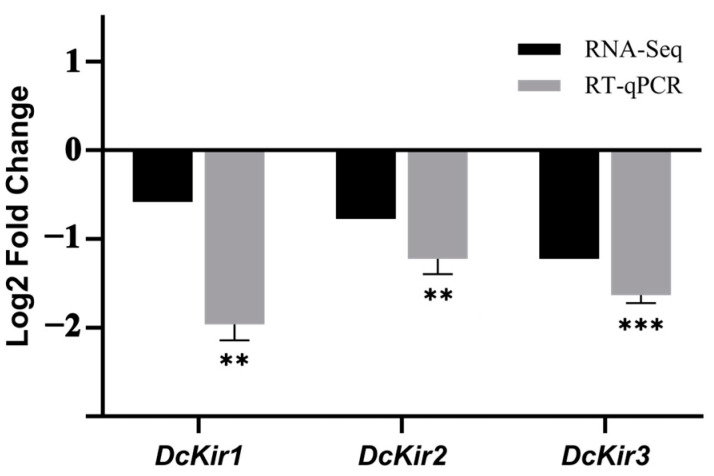
Comparison of expression levels of *DcKirs* by RT-qPCR and RNA-Seq. β-actin was used as the reference gene for RT-qPCR normalization. The data are represented as the mean ± SEM (n = 3). mRNA expression levels for the selected genes were calculated using the 2^−ΔΔCT^ method. Student’s *t* tests were performed for comparison with the control group, ** *p* < 0.01, and *** *p* < 0.001.

Previous research suggests that flonicamid may specifically target Kir channels. Even at nanomolar concentrations, flonicamid can block the Kir channels in *N. lugens*, effectively inhibiting their activity [11]. Among the DEGs of the ion channel activity pathway, we identified one Kir channel gene (Dcitr10g10250.1). To validate the results from the sequencing data, we identified two other Kir genes of the *D. citri* from the transcriptome, qRT-PCR was used to analyze three genes involved in Kir channels after the flonicamid treatment. Validation results indicate that the transcript levels of the tested *DcKirs* followed the trend of the transcriptome data, and all tested genes were significantly down-regulated (Figure 3).

### 3.5. Molecular Characteristics of DcKirs

**Figure 4:**
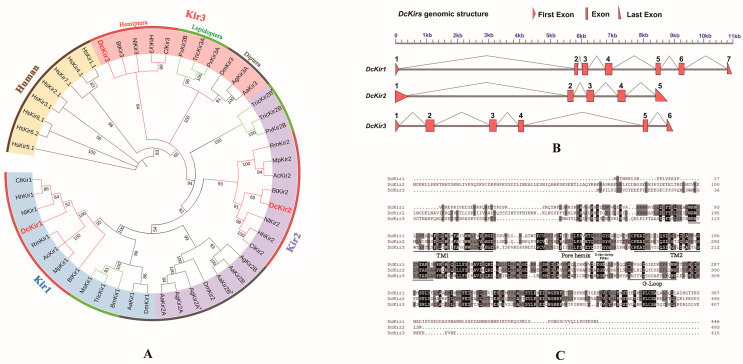
Phylogenetic analysis, genomic structure and amino acid alignments of *DcKirs*. (**A**) Phylogenetic analysis of *Kirs* from *D. citri* and other species. The *DcKirs* are indicated with red color. Accession numbers are listed in Table S4. (**B**) Genomic structures of *DcKirs*. The reds represent the exons, and the gray lines represent the original genomic scaffold sequences. (**C**) Amino acid alignments of *Kirs* from *D. citri*. The underlined lines consist of conserved domains, including the G-Loop, ion selectivity filter, pore helix, and transmembrane domains (TM1 and TM2).

Three Kir channel genes were successfully cloned from *D. citri*, which were identified as *DcKir1* (GenBank: PP315634), *DcKir2* (GenBank: PP315635), and *DcKir3* (GenBank: PP315636) based on NCBI BLAST results and phylogenetic analyses (Figure 4A). The open reading frames of the *DcKir1*, *DcKir2* and *DcKir3* were 1341 bp, 1497 bp, and 1170 bp, respectively. The phylogenetic analysis of *DcKirs* from Hemipteran, Dipteran, Lepidopteran, and human Kirs revealed that insect Kirs were divergent from human Kirs. The Hemipteran Kirs were grouped and classified into three subclades (Kir1, Kir2, Kir3) and there is only one member for the Kir1, Kir2, and Kir3 subfamily in *D. citri*. Hemipteran Kirs were clustered with their orthologs in Lepidopteran and Dipteran and were more closely related to Lepidopteran Kir orthologs.

The analysis of genomic positions and structures showed that the length of the *DcKir1* genomic sequence is 11.0 kilobases (kb) and it consists of five exons. The length of the *DcKir2* genomic sequence is 8.7 kb and it consists of five exons. The length of the *DcKir3* genomic sequence is 8.9 kb and it consists of six exons (Figure 4B). The deduced amino acid sequences were compared, and it was found that all of the *DcKir* proteins exhibited the characteristic structural motifs commonly found in the Kir channel family, including the ion selectivity filter, pore helix, two transmembrane domains, and G-Loop (Figure 4C).

### 3.6. Developmental and Tissue Distribution of DcKirs

**Figure 5:**
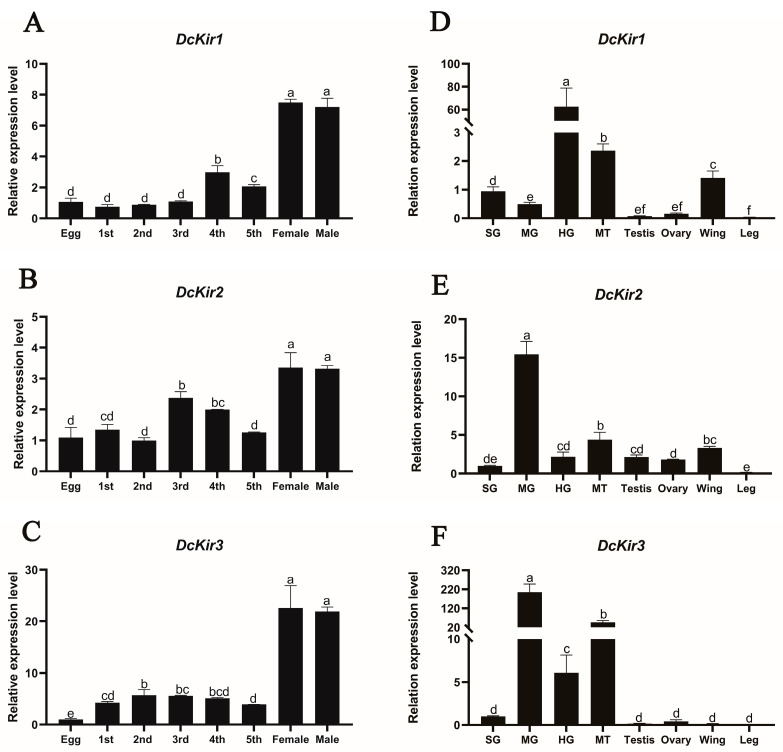
The expression patterns of *DcKirs* in different developmental stages (**A**–**C**) and tissues (**D**–**F**). Different developmental stages of *D.citri*, including eggs, 1st to 5th instar nymphs, and adults (1-day-old females and 1-day-old males). Different tissues are derived from 1-day-old adults. Tissue abbreviations: salivary gland, SG; midgut, MG; hindgut, HG; and Malpighian tubules, MT. Error bars indicate ± SEM of three biological repeats. The different letters on the column indicate significant differences (*p* < 0.05; one-way ANOVA, LSD test).

The expression patterns of *DcKirs* in different tissues and developmental stages were investigated using RT-qPCR. The determination of the expression profiles of *DcKirs* in different developmental stages of *D. citri* showed that the expressions of all these genes were significantly increased after *D. citri* eclosion into the adults and *DcKir3* had higher expressions noticeably in nymph and adult stages compared to *DcKir1* and *DcKir2* (Figure 5A–C).

The results of tissue distribution analysis found that *DcKirs* in the legs showed extremely low expression levels (Figure 5D–F). The relative expression of *DcKir1* in the hindgut was observed to be 67.6, which was 65.7, 130.5, 26.4, 331.3, 372.7, 44.3 and 1545.2 times higher than that in salivary glands, midgut, Malpighian tubules, ovaries, testes, wings, and legs, respectively. The expression of the *DcKir1* was significantly lower in the midgut than that of *DcKir2* and *DcKir3*. *DcKir2* expression was notably higher in the midgut than in other tissues, followed by the Malpighian tubules and wings. The *DcKir3* was also highly expressed in the midgut, like *DcKir2*, and it showed significantly high expression in Malpighian tubules too. Lower expression levels of *DcKir3* were found in other tested tissues including ovaries, testes, wings, and legs.

## 4. Discussion

The Asian citrus psyllid, *Diaphorina citri* Kuwayama, is the main natural vector insect of the citrus Huanglongbing (HLB) [11]. Currently, the effective control of field populations of *D. citri* is the main way to interrupt the spread of HLB. However, *D. citri* has developed high resistance to common insecticides due to frequent and excessive use of broad-spectrum chemical insecticides like organophosphate, pyrethroid, and neonicotinoid [18,23]. Flonicamid is a selective systemic insecticide belonging to a new class of insecticides with no cross-resistance to other commonly used insecticides and high activity against piercing–sucking mouthparts [11,24]. The study demonstrated that the LC_50_ of flonicamid on *Myzus persicae*, *Aphis gossypii*, *Rhopalosiphum padi*, *Schizaphis graminum*, and *Lipaphis erysimi*, after 5 days of treatment, ranged from 0.6 to 2.0 mg AI L^−1^. Additionally, it was found that flonicamid inhibited the feeding behavior of aphids within 0.5 h [9]. The susceptibility of different *N. lugens* populations to flonicamid was similar, with LC_50_ values of 44.6 and 46.0 mg AI L^−1^ at 5 d post dose [11]. LC_50_ values for flonicamid-treated cotton leafhoppers ranged from 358.1 mg AI L^−1^ at 4 days to 239.2 mg AI L^−1^ at 5 days [19]. However, in Lepidoptera, flonicamid showed no insecticidal activity against *Chilo suppressalis* larvae. The mortality rate of *C. suppressalis* larvae treated with a concentration of 1200 mg AI L^−1^ for seven days was very low. Moreover, the guts of the treated larvae were empty and shriveled when compared to the control [10]. However, the effectiveness of flonicamid against *D. citri* has not been studied. In this study, we found that the LC_50_ of flonicamid-treated *D. citri* adults for 4 d was 16.6 mg AI L^−1^. The dissection of the gut revealed a shriveled midgut and inconsistent coloration of the Malpighian tubules, symptoms potentially attributed to the flonicamid inhibition of *D. citri* feeding.

Insect ion channels play a crucial role in transmitting information between cells and regulating cellular action potentials, making them important targets for various insecticides. For instance, sodium channels in insects play a crucial role in generating and conducting action potentials, which is why they are targeted by organochlorine and pyrethroid insecticides [25]. Similarly, Gamma-aminobutyric acid-gated chloride channel (GABA-Cl) is the binding site of insecticides such as pyrethroid, organophosphate, and cyclodiene insecticides [26]. A previous study had also identified Kir channel as a potential target of flonicamid [11]. Although the molecular target of flonicamid has been explored in other species, recent findings have added complexity to our understanding. A recent study identified nicotinamidase (Naam) as the primary molecular target of flonicamid in *Drosophila* [12], suggesting that flonicamid may have multiple modes of action depending on the insect species and physiological context. However, the role of ion channels, particularly potassium channels, in flonicamid’s action on *D. citri* remains underexplored. This study analyzed gene expression differences in *D. citri* treated with flonicamid by transcriptomic sequencing and identified a total of 345 DEGs, including 136 up-regulated genes and 209 down-regulated genes. Interestingly, six genes related to ion channel activity were found to be down-regulated by flonicamid, suggesting that flonicamid has an inhibitory effect on the activity of ion channels.

Studies have reported that Kir inhibitors can disrupt the normal secretion activity of Malpighian tubules, resulting in a lethal effect on insects, highlighting the potential of Kir as a new target for insecticides [27]. Among the six differentially expressed genes in ion channel activity after flonicamid treatment, we identified one Kir gene (Dcitr10g10250.1) and then cloned and identified all *DcKirs* from the transcriptome. In Hemiptera, there are three Kir genes (*Kir1*, *Kir2*, *Kir3*) in the genomes of *D. citri* and *N. lugens* [11], whereas aphids have only two Kir genes and lack *Kir3* [20]. In Diptera, there is a duplication of *Kir2* and *Kir3*, as observed in mosquitoes such as *Anopheles gambiae* and *Aedes aegypti* [28]. However, In Lepidoptera, Kir2 and Kir3 have evolved into two distinct subtypes (subtype A and subtype B). Five Kir genes (*Kir1*, *Kir2A*, *Kir2B*, *Kir3A*, *Kir3B*) are expressed in the *C. suppressalis*, and a *Kir2* subtype previously identified as *Kir4* has been identified in *Plutella xylostella*. Phylogenetic analysis shows that Hemiptera Kirs cluster with their orthologs in Lepidoptera and Diptera, with a closer relationship to the orthologs of Lepidoptera Kirs [10,14,15].

Kir mRNAs are dynamically expressed during development and widely expressed in insect tissues [14,29]. Kir has been reported to be involved in many physiological functions of insects, such as Malpighian tubules excretion [30,31], salivary secretion and feeding [11], reproduction and development [32], wing differentiation [33], and so on. After *D. citri* eclosion to an adult, the expression of *DcKirs* was significantly increased, especially *DcKir3*. *DcKirs* were highly expressed mainly in the gut tissues: *DcKir1* was mainly expressed in the hindgut, *DcKir2* was mainly expressed in the midgut, and *DcKir3* was significantly highly expressed in both the midgut and the Malpighian tubules. *DcKir1* and *DcKir2* showed relatively high expression levels in the wings of *D. citri*, whereas their expression was almost undetectable in the legs. In the ovaries, the relative expression levels of *DcKirs* are notably low. This pattern contrasts with other species, such as *C. suppressalis* and *A. aegypti*, where *Kir1* and *Kir2A* exhibit relatively high expression levels in ovaries, while other Kir genes are minimally expressed [10,34]. In the salivary glands of *N. lugens*, *NlKir1* and *NlKir2* are highly expressed, while Kir genes show low expression in *D. citri* [11]. These data suggest that Kirs may play an important role in the functions of digestion and metabolism, and wing development in *D. citri*, and may be related to the observed phenotype of midgut shrinkage in *D. citri* treated with flonicamid. The low expression of *DcKirs* in the ovaries and salivary glands suggests that *D. citri* may use alternative ion channels to adapt to its reproductive and feeding habits. Alternatively, *D. citri* may exhibit tissue-specific adaptations in Kir channel expression due to unique evolutionary pressures.

## Figures and Tables

**Table 1:** Toxicity of flonicamid in *D. citri* adult.

Insecticide	n ^1^	Regression Equation ^2^	LC_50_ (95% CL) ^3^ (mg AI L^−1^)	R^2^	Df ^4^
Flonicamid	200	y = 1.88 + 0.67 x’	16.635 (12.501–23.930)	0.998	3

^1^: Total number of adults (n) tested was 200 individuals. ^2^: The regression equation used was x’ = logarithm in base 10 of the covariate (X). ^3^: The confidence limit (CL) for lethal concentration (LC) values was 95%. ^4^: Degree of freedom (df) was calculated by probit analysis (SPSS version 26.0).

## Data Availability

Data are contained within the article or in the Supplementary Materials. The mRNA data have been uploaded to NCBI (GenBank accession number: PRJNA1114007). Further inquiries can be directed to the corresponding author.

## Supplementary Materials

The following supporting information can be downloaded at: https://www.mdpi.com/article/10.3390/insects15110900/s1. Table S1: Primers used for clone and expression analysis of genes in *Diaphorina citri*; Table S2: Summary of the transcriptome data; Table S3: The statistics of reads mapped to the reference genome; Table S4: Accession numbers of Kir subunit amino acid sequences analyzed in Figure 4A.
