# Supplementary material for: Toxicity of Flonicamid to Diaphorina citri (Hemiptera: Liviidae) and Its Identification and Expression of Kir Channel Genes
Source: Insects. 2024 Nov 18;15(11):900. doi: 10.3390/insects15110900 (PMC11594753; doi:10.3390/insects15110900)
Supplement: Supplementary file 1 [file insects-15-00900-s001.zip › Supplementary File .pdf]

Table S1. Primers used for clone and expression analysis of genes in *Diaphorina citri*

Gene	Primer Names	Primer Sequences	TM (℃)	Purpose
DcKir1	*DcKir1*-F	TATGGCGGTGAGCAATAGCG	50	DNA cloning
	DcKir1-R	GGCACAGAGACAAAGGAAGCC		
DcKir2	*DcKir2*-F	CGGAGCACTCGCTAATTCCA	48	
	DcKir2-R	CATGCGGACATTTTCAGCCG		
DcKir3	*DcKir3*-F	TGTAGACGCTCTGGCAACAC	55	
	DcKir3-R	TCCAAAGGCCACCAGATGAC		
Actin	*Actin*-F	CCATCTTGGCTTCTCTGTCTAC	60	Real-time Quantitative PCR
	Actin-R	CATTTGCGGTGAACGATTCC		
DcKir1	*DcKir1*-q-F	CCTGTGGGAATGGAACCGAA	60	
	DcKir1-q-R	GGATCGGGATAGGTCTCGC		
DcKir2	*DcKir2*-q-F	CCAACACGCCAAGAGCAAAA	60	
	DcKir2-q-R	CGAAAAGTTCCTCCTCCGCT		
DcKir3	*DcKir3*-q-F	GGCGAGTGTTTCTTTTCCGT	60	
	DcKir3-q-R	TTCTGGAACATGGCGTTCGT		

Table S2. Summary of the transcriptome data

Sample	Raw reads	Clean reads	Clean data (bp)	Q20 (%)	Q30 (%)	GC (%)
CK1	43059388	42835710	6383654311	97.62	93.36	42.02
CK2	56059322	55799910	8308751807	97.81	93.84	41.85
CK3	47812588	47558272	7085455796	97.57	93.35	41.77
Flonicamid-1	56143268	55862122	8330010669	97.75	93.75	42.18
Flonicamid-2	61083282	60773960	9059160629	97.65	93.51	42.26
Flonicamid-3	43145686	42909466	6397517929	97.62	93.44	41.92

Table S3. The statistics of reads mapped to the reference genome

Sample	Total reads	Total mapped	Multiple mapped	Uniquely mapped
CK1	41151522	34919109 (84.85%)	10261039 (24.93%)	24658070 (59.92%)
CK2	53521952	45379356 (84.79%)	13424573 (25.08%)	31954783 (59.70%)
CK3	45430958	38254241 (84.20%)	11449332 (25.20%)	26804909 (59.00%)
Flonicamid-1	53144638	45145941 (84.95%)	13706255 (25.79%)	31439686 (59.16%)
Flonicamid-2	57956458	49708782 (85.77%)	14772426 (25.49%)	34936356 (60.28%)
Flonicamid-3	40673860	34490958 (84.80%)	10254192 (25.21%)	24236766 (59.59%)

Table S4. Accession numbers of Kir subunit amino acid sequences analyzed in Figure 4A

Abbreviation	Species	NCBI Accession #	Abbreviation	Species	NCBI Accession #
AaKir1	*Aedes aegypti*	AAEL008932	HsKir1.1	*Homo sapiens*	NP_000211
AaKir2A	*Aedes aegypti*	AAEL008928	HsKir2.1	*Homo sapiens*	AAF73241
AaKir2B	*Aedes aegypti*	AAEL008931	HsKir3.1	*Homo sapiens*	AAH22495
AaKir2B'	*Aedes aegypti*	AAEL013373	HsKir4.1	*Homo sapiens*	AAB07046
AaKir3	*Aedes aegypti*	AAEL001646	HsKir5.1	*Homo sapiens*	AAG09401
AcKir1	*Acyrthosiphon pisum*	XP_003246666	HsKir6.1	*Homo sapiens*	AAH00544
AcKir2	*Acyrthosiphon pisum*	BAH71694	HsKir6.2	*Homo sapiens*	AAI12359
AgKir2A	*Anopheles gambiae*	AGAP001281	HsKir7.1	*Homo sapiens*	AAH37290
AgKir2A'	*Anopheles gambiae*	AGAP001283	MpKir1	*Myzus persicae*	XP_022179458
AgKir2B	*Anopheles gambiae*	AGAP001284	MpKir2	*Myzus persicae*	XP_022159991
AgKir3A	*Anopheles gambiae*	AGAP007818	MsKir1	*Manduca sexta*	XP_030028772
BmKir1	*Bombyx mori*	XP_012551257	NlKir1	*Nilaparvata lugens*	ASN63883
BtKir1	*Bemisia tabaci*	XP_018903156	NlKir2	*Nilaparvata lugens*	ASN63884
BtKir2	*Bemisia tabaci*	XP_018903159	NlKir3	*Nilaparvata lugens*	ASN63885
BtKir3	*Bemisia tabaci*	XP_018914499	PxKir2B	*Plutella xylostella*	XP_011553023
ClKir1	*Cimex lectularius*	XP_014261722	PxKir3A	*Plutella xylostella*	XP_011566889
ClKir2	*Cimex lectularius*	XP_24081293	PxKir3B	*Plutella xylostella*	XP_011562711
ClKir3	*Cimex lectularius*	XP_014248345	RmKir1	*Rhopalosiphum maidis*	XP_026822661
DmKir1	*Drosophila melanogaster*	NP_1262861	RmKir2	*Rhopalosiphum maidis*	XP_026808359
DmKir2	*Drosophila melanogaster*	ACL85253	TricKir1	*Trichoplusia ni*	XP_026724912
DmKir3	*Drosophila melanogaster*	NP_001137835	TricKir2B	*Trichoplusia ni*	XP_026724920
HhKir1	*Halyomorpha halys*	XP_014273294	TricKir2B’	*Trichoplusia ni*	XP_026725705
HhKir2	*Halyomorpha halys*	XP_014273296	TricKir3A	*Trichoplusia ni*	XP_026735442
HhKir3	*Halyomorpha halys*	XP_024220073			
